# Peripherally Induced Oromandibular Dystonia Following Orthognathic Surgery

**DOI:** 10.7759/cureus.93629

**Published:** 2025-10-01

**Authors:** Kazuya Yoshida

**Affiliations:** 1 Department of Oral and Maxillofacial Surgery, National Hospital Organization, Kyoto Medical Center, Kyoto, JPN

**Keywords:** botulinum toxin therapy, jaw deformity, oromandibular dystonia, orthognathic surgery, peripherally induced movement disorder

## Abstract

Background: Peripherally induced movement disorders are hyperkinetic conditions triggered by peripheral trauma or surgical intervention. Oromandibular dystonia (OMD), a focal dystonia affecting the masticatory and lingual muscles, is the most common peripherally induced movement disorder within the stomatognathic system. However, OMD following orthognathic surgery has rarely been reported.

Objective: This study aimed to describe the clinical characteristics, latency, and treatment outcomes of patients who developed OMD after orthognathic surgery in a retrospective single-center case series of six patients.

Methods: This retrospective case series included patients presenting with involuntary orofacial movements after orthognathic surgery at Kyoto Medical Center between 2007 and 2025. Inclusion criteria were as follows: onset of OMD within three months following surgery, anatomical correlation, and patient-reported causality. Clinical features, surgical history, latency, and treatment outcomes were analyzed.

Results: Six patients (mean age 35.2 years) met the criteria. Four had mandibular prognathism, one had mandibular prognathism with tongue hypertrophy, and one had bimaxillary prognathism with open bite. All underwent sagittal split ramus osteotomy: two had additional Le Fort I osteotomy, one underwent intraoral vertical ramus osteotomy, and one had tongue reduction surgery. OMD subtypes included jaw closing (n = 2), tongue (n = 2), jaw opening (n = 1), and jaw deviation (n = 1). Mean latency to OMD onset was 39.5 days, while the mean delay before referral was 40.3 months. All patients were treated with botulinum toxin injections, resulting in symptomatic improvement. Notably, none of the surgeons initially recognized the association with surgery.

Conclusions: OMD may occur as a possible peripherally induced sequela of orthognathic surgery. Although rare, it is likely underdiagnosed. Oral and maxillofacial surgeons should remain aware of this potential complication to facilitate timely diagnosis and appropriate management.

## Introduction

Peripheral injury has been implicated in the development of various movement disorders, including dystonia, tremor, and parkinsonism [1,2]. Peripherally induced movement disorder is defined by a close temporal and topographic association between peripheral trauma and subsequent involuntary movements [3].

Within the stomatognathic system, peripherally induced movement disorders include oromandibular dystonia (OMD), orolingual dyskinesia, hemimasticatory spasm, and functional movement disorder [4,5]. OMD is the most frequent, characterized by sustained or task-specific contractions of the jaw, lingual, or perioral muscles, impairing chewing, swallowing, and speech [6-8].

Orthognathic surgery is commonly performed to correct jaw deformities, improve occlusion, and enhance aesthetics [9]. Well-documented complications include swelling, bleeding, infection, neuropathy, and facial nerve damage [9-11]. However, OMD following orthognathic surgery has rarely been reported. In fact, only a few isolated cases have been described in the literature [7].

Given the limited awareness among oral surgeons, this retrospective study aimed to describe six patients who developed OMD following orthognathic surgery, highlighting clinical features, latency, and treatment outcomes.

## Materials and methods

This was a retrospective, single-center case series conducted at Kyoto Medical Center between January 2007 and March 2025. During this period, approximately 3,000 patients with orofacial movement disorders were evaluated in our department. Among them, six patients fulfilled the criteria for peripherally induced OMD following orthognathic surgery.

Inclusion criteria were as follows: (1) onset of OMD within three months after orthognathic or related surgical procedures; (2) a clear topographic relationship between the surgical site and the distribution of dystonic symptoms; and (3) patient attribution of the onset of symptoms to surgery. Exclusion criteria included secondary dystonia due to central nervous system disease, psychogenic disorders, or onset of symptoms beyond three months postoperatively.

Study parameters included patient demographics, type of jaw deformity, type of surgical procedure, latency from surgery to OMD onset, duration from onset to referral, OMD subtype, and treatment outcomes. Details of botulinum toxin therapy, including the number of sessions, representative dosage ranges per muscle (e.g., masseter 20-50 U, genioglossus 20-50 U), and specific target muscles, were also documented.

**Figure 1:**
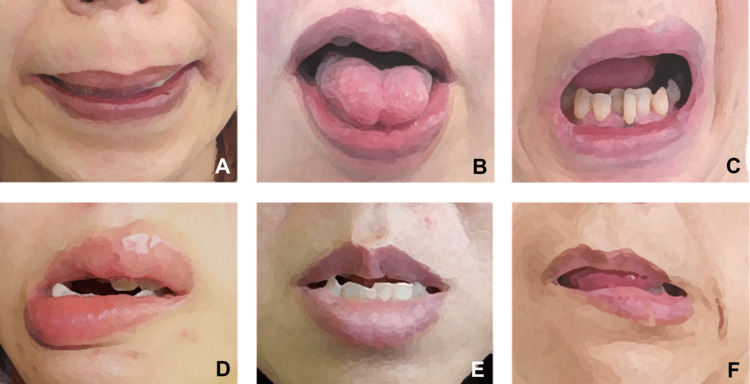
Schematic diagram of OMD subtypes (A) Jaw closing dystonia, (B) tongue dystonia, (C) jaw opening dystonia, (D) jaw deviation dystonia, (E) jaw protrusion dystonia, (F) lip dystonia. OMD: oromandibular dystonia. Image Credit: Author's original creation, illustrated using Adobe Illustrator (Adobe Inc., San Jose, CA, USA).

Diagnosis was based on established clinical criteria, including sustained, repetitive, and task-specific contractions of the orofacial muscles [8,12,13]. Subtypes were categorized as jaw closing, tongue, jaw opening, jaw deviation, jaw protrusion, or lip dystonia (Figure 1). All patients underwent botulinum toxin therapy tailored to the affected muscles [8,14].

The study was approved by the Institutional Review Board and Ethics Committee of Kyoto Medical Center (Approval No. 15-031). Given the retrospective design, the need for written informed consent was waived.

## Results

A total of six patients (four women and two men) were identified who developed OMD after orthognathic surgery during the study period. The mean age at the time of surgery was 31.5 years, and the mean age at OMD onset was 35.2 years. With regard to jaw deformities, four patients presented with mandibular prognathism, one had mandibular prognathism with tongue hypertrophy, and one had bimaxillary prognathism with open bite.

All patients underwent sagittal split ramus osteotomy (SSRO) as part of their surgical treatment. Two of these patients also underwent Le Fort I osteotomy, one underwent intraoral vertical ramus osteotomy (IVRO), and one underwent tongue reduction surgery. The subtypes of OMD observed were jaw closing (n = 2), tongue (n = 2), jaw opening (n = 1), and jaw deviation (n = 1) (Table 1).

**Table 1:** Types of jaw deformities and surgical procedures of patients with peripherally induced OMD after orthognathic surgery OMD: oromandibular dystonia, SSRO: sagittal split ramus osteotomy, IVRO: intraoral vertical ramus osteotomy, Le Fort: Le Fort I osteotomy.

Patient	Age (years)	Sex	Types of jaw deformities		Surgical procedures	Age at operation (years)
							1	32	Woman	Mandibular prognathism		SSRO + IVRO	22
2	22	Man	Mandibular prognathism		SSRO	20
3	40	Woman	Bimaxillary prognathism		Le Fort + SSRO	35
4	48	Woman	Mandibular prognathism, tongue hypertrophy		SSRO, tongue reduction surgery	46
							5	40	Woman	Open bite		Le Fort + SSRO	38
6	29	Man	Mandibular prognathism		SSRO	28
Mean ± SD	35.2 ± 9.3	-	-		-	31.5 ± 10

The mean latency from surgery to the onset of dystonic symptoms was 39.5 days (range, 14-82 days) (Table 2). Despite the relatively short latency, the mean delay before referral to our center was 40.3 months, reflecting a substantial diagnostic gap. Notably, none of the operating surgeons initially acknowledged a potential causal relationship between the surgery and the subsequent dystonia.

**Table 2:** Summary of treatment for patients with peripherally induced OMD after orthognathic surgery OMD: oromandibular dystonia.

Patient	Subtype of OMD	Duration of OMD (months)	Interval of OMD onset after surgery (days)	Botulinum toxin therapy (times)	Injected muscles
						1	Jaw closing dystonia	114	30	3	Bilateral masseter, temporalis, and medial pterygoid muscles
												2	Tongue dystonia	24	21	5	Bilateral genioglossal muscles
						3	Jaw opening dystonia	52	60	2	Bilateral lateral pterygoid, mentalis, and orbicularis oris muscles
4	Tongue dystonia	20	42	11	Bilateral genioglossal muscles						
5	Jaw closing dystonia	20	56	2	Bilateral masseter muscles
6	Jaw deviation dystonia	12	28	13	Bilateral lateral pterygoid muscles
Mean ± SD	-	40.3 ± 38.6	39.5 ± 15.9	6 ± 4.8	-

All six patients were treated with botulinum toxin therapy, which was tailored to the specific muscles involved. The targeted muscles included the masseter, temporalis, lateral and medial pterygoids, genioglossus, and orbicularis oris (Table 2). Patients received an average of six botulinum toxin therapy injection sessions, and all experienced clinically meaningful improvement in symptoms, with reductions in involuntary movements and functional impairment. No severe adverse effects were reported. 

## Discussion

This case series highlights the occurrence of OMD as a possible peripherally induced movement disorder following orthognathic surgery. Although OMD has long been recognized after various dental and oral surgical procedures, its development specifically after orthognathic surgery has been rarely documented. The temporal proximity and anatomical relevance observed in our six patients suggest a possible clinically meaningful association between surgical intervention and subsequent dystonic symptoms.

Orthognathic surgery is widely regarded as safe and effective, but like all major surgical interventions, it carries a spectrum of risks. Well-documented complications include infection, relapse, temporomandibular joint symptoms, hemorrhage, neurosensory disturbances, and facial nerve damage [9-11]. While these represent commonly recognized complications, our findings suggest that OMD, although extremely rare, may also arise as a significant postoperative sequela.

The mechanisms underlying peripherally induced dystonia remain incompletely understood. The widely cited “two-hit hypothesis” posits that peripheral trauma may trigger dystonia in predisposed individuals [15]. Orthognathic surgery entails major changes in occlusion, temporomandibular joint mechanics, and trigeminal sensory input, all of which may induce maladaptive neuroplasticity within central motor circuits. Such aberrant reorganization can lower the threshold for dystonic activity. Interestingly, tongue and jaw-opening dystonia emerged in patients who had undergone more complex procedures, such as tongue reduction or bimaxillary osteotomies, suggesting that surgical complexity and invasiveness may contribute to increased susceptibility [16].

When recommending preoperative counselling and evaluating outcomes, it is important to consider functional and quality-of-life measures. A recent systematic review demonstrated that patient satisfaction following orthognathic treatment is closely tied to functional and aesthetic outcomes [17]. This further supports our recommendation that OMD, although rare, should be included in risk discussions with patients.

All patients in this series improved with botulinum toxin therapy, reaffirming its role as the treatment of choice for OMD [8,14]. Injection strategies varied depending on the dystonia subtype and involved not only masticatory muscles but also lingual and perioral muscles. Accurate targeting is critical for success.

This study has several limitations. First, the small sample size and single-center design limit generalizability. Second, the retrospective design precluded standardized pre- and postoperative neurological assessments. Third, referral and selection bias may have influenced case identification. Despite these limitations, this study provides important clinical insight into an underrecognized complication of orthognathic surgery.

## Conclusions

OMD may occur as a peripherally induced sequela of orthognathic surgery. Awareness among oral surgeons is essential for early recognition, patient education, and timely referral for treatment.
